# Cancer, Fertility Preservation, and Future Pregnancy: A Comprehensive Review

**DOI:** 10.1155/2012/953937

**Published:** 2012-03-18

**Authors:** Michelle L. Matthews, Bradley S. Hurst, Paul B. Marshburn, Rebecca S. Usadi, Margaret A. Papadakis, Terry Sarantou

**Affiliations:** ^1^Department of Obstetrics and Gynecology, Carolinas Medical Center, P.O. Box 32861, Charlotte, NC 28232, USA; ^2^Blumenthal Cancer Center, Carolinas Medical Center, 1025 Morehead Medical Drive, Charlotte, NC 28204, USA

## Abstract

Given the increases in 5-year cancer survival and recent advances in fertility preserving technologies, an increasing number of women with cancer are presenting for discussion of fertility preserving options. This review will summarize the risk of infertility secondary to cancer treatment, available treatment options for fertility preservation, and techniques to reduce future risks for patients. Concerns that will be addressed include the risk of the medications and procedures, the potential delay in cancer treatment, likelihood of pregnancy complications, as well as the impact of future pregnancy on the recurrence risk of cancer. Recent advances in oocyte cryopreservation and ovarian stimulation protocols will be discussed. Healthcare providers need to be informed of available treatment options including the risks, advantages, and disadvantages of fertility preserving options to properly counsel patients.

## 1. Introduction

An estimated 1 out of 47 women will be diagnosed with some type of invasive cancer by age 40 years, and approximately 774,370 women will be diagnosed with cancer in 2011 [1]. The most common cancers in reproductive age women are breast, melanoma, cervical cancer, non-Hodgkin's lymphoma, and leukemia [2]. Fortunately, the 5-year relative survival for all cancers is up from 50% (1975–1977) to 68% (1999–2006) reflecting improved diagnosis and treatment. The 5-year female cancer survival is dependent on stage at diagnosis but is currently 90% for breast, 91% for melanoma, 71% for cervical, 69% non-Hodgkin lymphoma, and 55% for leukemia [1]. Given the relatively high incidence of cancer in reproductive age women and improvements in 5-year survival, an increasing number of women are presenting for discussion of fertility preservation and pregnancy after cancer treatment.

 Fertility preservation is a rapidly evolving field that includes medical and surgical treatments to decrease the impact of cancer treatments on future fertility. Traditional fertility preserving techniques for patients undergoing radiation treatment included pelvic shielding or surgical repositioning of the ovaries out of the pelvis. Medical treatments to suppress ovarian function during chemotherapy have also been reported to decrease the effect on cancer treatments on future ovarian function. These modalities still rely on residual ovarian function after cancer treatments to conceive. Newer techniques to preserve ovarian reserve, oocytes, and embryos prior to cancer treatments have been developed to provide an opportunity to conceive in the event that cancer treatments result in permanent loss of ovarian function.

This review will summarize available treatment options for fertility preservation in cancer patients. Concerns that will be addressed include the risk of the medications and procedures, the potential delay in cancer treatment, as well as the impact of future pregnancy on the recurrence risk of cancer. Healthcare providers need to be aware of available treatment options including the risks, advantages, and disadvantages of fertility-preserving options to properly counsel patients.

## 2. Methods and Materials

 We performed a MEDLINE search to July 2011 using the following terms: fertility preservation, cancer, in vitro fertilization, assisted reproduction, ovarian stimulation, oocyte vitrification, ovarian preservation, hereditary cancer, childhood cancer, preimplantation genetic diagnosis, ovarian reserve, pregnancy, and cancer. Studies included in this review include publications in peer-reviewed journals.

## 3. Results and Discussion

### 3.1. Counseling Patients on Future Fertility Prior to Cancer Therapy

 Future fertility is a significant concern for patients undergoing cancer treatment. Studies have shown that the psychological impact of cancer-related infertility is substantial with 77% of patients reporting clinically significant levels of distress in relation to loss of fertility [3]. A survey of over 600 women with breast cancer indicated that 73% of women reported some degree of concern about the possibility of becoming infertile after treatment and 29% of patients indicated that their desire for future fertility impacted their cancer treatment decisions. In fact, many women indicated that they may choose a less toxic dose of chemotherapy to help preserve fertility even if it may increase the risk of cancer recurrence [4]. Cancer survivors also have higher depression and distress scores if they have unmet informational needs about future reproductive options [5]. Recognizing these concerns, the American Society of Clinical Oncology (ASCO) published recommendations in 2006 on fertility preservation in cancer patients. These guidelines state that oncologists should address the possibility of infertility with cancer patients and be prepared to discuss possible fertility preservation options or refer the patient to a reproductive specialist [6].

 Despite the 2006 ASCO recommendations, a nationwide survey of oncologists in 2009 reported that less than 50% referred patients to a reproductive specialist [7]. A survey of academic medical centers reported similar results with less than 40% referring patients to a reproductive specialist although 95% reported that they routinely discussed the effect of cancer treatment on fertility [8]. Factors that were associated with a higher likelihood to refer in patients with breast cancer included patients with a family history of breast cancer, older age, early stage cancer, and receiving care at an academic center [9]. Interestingly, a review of NIH intramural clinical trials for pediatric cancer, gynecologic cancer, or for stem-cell transplantation found that only 47% of patient consents addressed future infertility risks after cancer treatment [10].

#### 3.1.1. Risk of Infertility after Cancer Treatment

 The adverse effects of chemotherapy and radiotherapy on female reproduction have long been recognized. Part of the difficulty in counseling patients regarding the risk of infertility and/or subsequent pregnancy complications is that the risks are dependent on several factors. These risks include the dose and duration of treatment, other risk factors for infertility, the age of the patient, and the patient's baseline ovarian reserve at the time of initiation of treatment.

 Pelvic and/or abdominal radiation impacts future fertility by affecting both uterine and ovarian function. Radiation is typically administered as external beam therapy (teletherapy), intracavitary (brachytherapy), or total body irradiation as is utilized with stem-cell transplantation. Radiation is most commonly used in children for treatment of Wilm's tumor, abdominal rhabdomyosarcoma, and Ewing's sarcoma of the pelvis or spine. The effects of radiation therapy are dependent on the dose and the field applied. Radiation is typically targeted at the affected area; however, the impact of scattered radiation during treatments is also a consideration. Total body irradiation as used in stem-call transplantation has an over 80% risk of permanent amenorrhea. Limited field external beam radiation has a reduced risk depending on the location, dose, fractionation schedule, and age of the patient at the time of radiation treatment. In a study of 2000 women treated with pelvic radiotherapy, 95% had permanent ovarian failure following radiotherapy of 5–105 Gy [11]. It was reported that radiation doses over 5 Gy for women over 30 years results in permanent amenorrhea, however, it has been reported more recently that the lethal dose (LD50) of the human oocyte is actually less than 2 Gy [12].

 Although the uterus is relatively resistant to the effect of radiotherapy, there is a concern that radiation may decrease uterine blood supply, volume, and endometrial thickness. Exposure to 20–30 Gy of abdominal or pelvic radiation has been shown to increase the future risk of miscarriage, preterm labor, and low birth weight [13]. The impact of radiation on future uterine function is dependent on the age at radiation in childhood cancers. The prepubertal uterus appears to be more vulnerable to the effects of radiation. Hormonal stimulation with estrogen and progesterone to improve endometrial thickness, and blood flow after radiation has been evaluated with variable effectiveness. The uterine volume increased significantly from 6.5 mL to 16.3 mL but was still less than controls after 3 months of hormonal replacement provided to patients with amenorrhea after radiation treatments. It was noted than patients exposed to prepubertal radiation had less improvement that patients exposed postpubertally [14]. Another study evaluating 3 childhood cancer survivors that received high-dose abdominal or pelvic radiation (30–54 Gy) found no increase in uterine volume, blood flow, or endometrial thickness with high-dose estrogen therapy [15]. These limited studies indicate that higher dosages of radiation affect uterine function and are most significant if administered prepubertally. Hormonal therapy may have limited benefit for improving endometrial development and patients that conceive should be considered at higher risk of preterm labor, delivery, and low-birth weight.

 The primary impact of chemotherapy on fertility is related directly to the loss of ovarian function secondary to the gonadotoxicity of many chemotherapeutic agents. Cell-cycle nonspecific alkylating agents such as cyclophosphamide will destroy resting primordial oocytes while antimetabolite agents (methotrexate) have limited effect on ovarian function. The greatest risk is in women over age 40 years receiving alkylating agents with up to 80% of patients having permanent amenorrhea after treatment. However, in women under 30 years, the risk of permanent amenorrhea is substantially decreased to less than 20% [6]. The effect of chemotherapy will also depend on whether it is radical or adjuvant, single agent, or combination. Fortunately, the more recent ABVD regimen (Doxorubicin, Bleomycin, Vincristine, and Dacarbazine) used in the treatment of Hodgkin's disease is significantly less toxic to fertility than the older MOPP (Mechlorethamine, Vincristine, Procarbazine, and Prednisilone). The classical CMF (Cyclophosphamide, methotrexate, 5-fluoruracil) regimen for breast cancer will result in over 70% amenorrhea rates for women over 40 years [16]. The newer Taxanes are still being evaluated for their impact on fertility but hopefully will be less gonadotoxic than currently used regimens. Unfortunately, estimates in the impact on fertility vary widely dependent on various factors, and, therefore, there is no definitive predictor prior to treatment making counseling on future fertility challenging for health care providers.

#### 3.1.2. Determining the Impact of Cancer Treatment on Ovarian Reserve

 The peak number of oocytes is found in females at 20 weeks of fetal life, and this number declines until menopause. The number of primordial follicles is approximately 500,000 at menarche; menopause occurs once that pool is nearly depleted. Although chronologic age is the most important predictor of oocyte quality and quantity, there is variability in the rate of ovarian aging. The term “ovarian reserve” is used to describe remaining ovarian oocyte quantity. Although menstrual cycles do not start to become irregular until a mean age of 45 to 55 years, endocrinologic changes associated with ovarian aging have been demonstrated for women age 35 to 40 years and at earlier ages after cancer treatment. Several modalities have been evaluated as markers of ovarian reserve including cycle day 2-3 FSH, antimullerian hormone (AMH), and ovarian ultrasound of antral follicles. Assessment of a patient's ovarian reserve both before and after cancer treatment may provide valuable information for patients in discussion of fertility preserving option prior to treatment and future fertility after treatment.

 Basal FSH values drawn during menstrual day 2-3 have been the routinely utilized as a marker of ovarian reserve. As FSH values increase, ovarian responsiveness decreases. An FSH value of 10–15 IU/L is generally considered borderline, and values over 15 IU/L are considered significantly elevated [17]. It is important when assessing FSH values to also evaluate basal estradiol as an elevation may suppress FSH and give a falsely reassuring value. A normal basal estradiol may vary between laboratories but typically is less than 60 pg/mL. FSH values may fluctuate widely between cycles particularly for patients with decreased ovarian reserve which limits its effectiveness as a marker of remaining ovarian function.

 AMH is a member of the transforming growth factor B family and is produced by the granulosa cells of the secondary, prenatal, and antral follicles. AMH levels decrease progressively until it becomes undetectable at menopause. Theoretically, this may be a better marker of ovarian reserve as it represents the number of early and developing follicles and appears to have less intercycle variability than FSH [18]. One significant advantage of this test is that it does not require assessment on cycle day 2 or 3 since there is limited variability during the menstrual. However, this test may not be routinely available at all laboratories, and no international standard has been developed yet for this assay.

 Ultrasound of ovarian follicle counts (AFC) has also been evaluated as a tool for predicting ovarian reserve. The number of antral follicles (<10 mm) present on a menstrual cycle day 2 to 3 by transvaginal ultrasound has also been correlated with other serum markers of ovarian function [19]. AFC is directly correlated with the number of oocytes retrieved during IVF and may prove to be the best predictor of ovarian reserve. Intercycle variability is present with all forms of ovarian reserve testing, and no single test has been consistently recommended. Basal FSH has been the mainstay of screening, but basal AFC and AMH may prove to be superior.

 There is limited information on the impact of cancer treatment on markers of ovarian reserve. Small studies in young cancer patients have indicated that FSH, AMH and AFC have all been demonstrated to change in response to chemotherapy [20]. One study evaluated 42 premenopausal women receiving neoadjuvant chemotherapy that were followed over 5 years. Pretreatment FSH, AMH, and AFC and all were found to reflect future ovarian activity for women with menses after chemotherapy, but AMH was the most predictive by logistic regression [21, 22]. Further research is needed to determine the impact of cancer treatments on markers of ovarian reserve and any correlation with future fertility. It is important to consider that most research has evaluated these markers in relation to success of ovarian stimulation for IVF; therefore, caution must be used in counseling patients on the likelihood of spontaneous pregnancy or with other fertility treatments.

### 3.2. Options for Fertility Preservation

#### 3.2.1. Ovarian Suppression during Chemotherapy

 It has been well documented that chemotherapeutic agents, particularly alkylating agents, have high levels of ovarian toxicity. Oocytes are contained in ovarian primordial follicles, and it is estimated that hundreds to thousands of these follicles initiate the maturation process each month and are susceptible to the gonadotoxic effects of chemotherapy. Primordial follicles are stimulated to initiate maturation through a complex process that is initiated by follicle stimulating hormone (FSH) release from the pituitary in response to hypothalamic gonadotropin releasing hormone (GnRH). Suppression of ovarian function through manipulation of GnRH has been evaluated as a mechanism to decrease the loss of primordial follicles.

 Administration of GnRH analogs results in downregulation of pituitary receptors within 10–14 days of administration and subsequent suppression of FSH release. This has been studied in animal models with promising results but data regarding effectiveness in humans is limited to small retrospective reports. A recent systematic review [23] evaluating the utility of GnRH agonists in patients with breast cancer summarized data on the 5 available nonrandomized studies. The largest study of 100 women receiving 12 months of GnRH analogs during cancer treatment found that 67% of patients recovered normal menses with 100% return of menstrual function for women less than 40 years of age. However, only 3 pregnancies were reported [24]. Smaller studies have reported resumption of menses for 72–90% of patients with several pregnancies reported.

 Unfortunately, it cannot be determined from these studies that the administration of GnRH agonists provided definitive ovarian protection. There are 4 reported ongoing prospective, randomized trials in women with hormone receptor-negative breast cancer to evaluate the effect on preserving fertility [25]. Outcome data from these studies will provide valuable information on the utility of this treatment in preserving ovarian function. It should be noted that there is some concerns regarding the use of GnRH agonists. It has been suggested that GnRH agonists may decrease the effect of tamoxifen if administered simultaneously and until more data is available, the ASCO recommends that women interested in this treatment receive it only as part of an approved clinical trial [6].

#### 3.2.2. Embryo Cryopreservation

 The basic principle of cryopreservation is to store cells or tissue for future use. Damage to cells during the cryopreservation process has been a barrier to the general use of this technology. Cryopreservation is typically performed by incubation in a low concentration of cryoprotectant to minimize ice crystal formation during freezing; however, cells with a high osmotic content such as oocytes are particularly vulnerable to damage. Embryos are composed of multiple blastomere cells and are more stable for cryopreservation. Due to the difficulties with oocyte cryopreservation, embryo cryopreservation has been the primary modality for fertility preservation and has been available since the 1980s. The most recent available data have indicated that over 21,000 embryo transfers occurred in USA in 2009 from frozen, thawed embryos resulting in per-cycle pregnancy rates of 35.6% 9 (<35 years), 30.9% (35–37 years), 26.8% (38–40 years), and 22.1% (40–42 years) with an average of approximately two embryos transferred per patient (http://www.sart.org/). 

Embryo banking has several advantages for patients interested in preserving fertility. It provides reassurance to a patient that she will have some potential to conceive if the cancer treatments result in permanent amenorrhea. There is also over 20 years of outcome data for cryopreserved embryos showing no effect on miscarriage, implantation rates, or live birth [26]. A disadvantage of embryo banking is the need to administer ovarian stimulation medications to obtain oocytes for fertilization. Ovarian stimulation is a particular concern for patients with hormonal sensitive tumors such as breast cancer and will be addressed further in this review.

 The American Society for Reproductive Medicine (ASRM) Ethics Committee published guidelines in 2005 on fertility preservation and reproduction in cancer patients. These guidelines state that the only established method of female fertility preservation is embryo cryopreservation and that experimental procedures such as oocyte or ovarian tissue cryopreservation should be offered only in a research setting with IRB oversight [27]. However, these recommendations may be revised with improvements in oocyte preservation technology and increasing numbers of live birth reported in the past several years.

#### 3.2.3. Oocyte Cryopreservation

 Recent advances in oocyte cryopreservation technology have expanded the use of this technology for fertility preservation. Disadvantages are similar to those of embryo banking including the risk of ovarian stimulation for patients with hormonally responsive cancers and the potential delay in starting cancer treatments. Oocyte banking is preferable over embryo banking for patients that do not have a partner and/or are not interested in utilizing donor sperm or have ethical concerns regarding cryopreservation of embryos.

 Until recently, the primary disadvantage of oocyte banking has been the lower success rate compared to embryo cryopreservation. The first pregnancy from oocyte cryopreservation was reported in 1986 [28], but few pregnancies were subsequently reported due to poor survival rates for oocytes. The poor survival rates for oocytes that have been cryopreserved and thawed are attributable to several factors. Oocytes have a relatively high volume compared to other cells and are susceptible to intracellular ice crystal formation. Cryopreservation of oocytes has also been shown to result in chromosome and DNA abnormalities as the meiotic spindle of oocytes is very sensitive to chilling. Oocytes are also more susceptible to damage from reactive oxygen species than other cells. Many of these parameters improve after fertilization, making embryos less susceptible to damage than oocytes [29].

 The more recent development of oocyte vitrification incorporates several modifications to traditional cryopreservation that result in less toxicity to oocytes. Oocyte vitrification exposes oocytes to higher concentrations of cryoprotectants for shorter durations of time followed by very rapid cooling. There have been over 500 pregnancies reported worldwide since 2005 with improvements in oocyte cryopreservation techniques [30]. A meta-analysis of randomized controlled trials assessing efficacy of oocyte vitrification reported similar fertilization, embryogenesis, and pregnancy from oocytes derived from vitrified oocytes compared to fresh oocytes. The authors state that increasing reports of successful cryopreservation of oocytes warrant reexamination of whether oocyte vitrification should still be considered an experimental technique [31].

#### 3.2.4. Ovarian Tissue Cryopreservation

 Ovarian tissue cryopreservation has also been evaluated as a modality to preserve future fertility. A portion of ovarian cortex is cryopreserved and then transplanted back to the pelvis, or other location (arm or abdominal wall has been reported) [32, 33]. The first report of an ovarian transplant operation occurred in 2000 [34] with the first pregnancy reported in 2004 [35] in a patient with non-Hodgkin's lymphoma. There have been fewer than 15 reported pregnancies worldwide with this technique; however, the first report of a woman that gave birth to a second child by natural conception after ovarian tissue transplantation has recently been reported [36].

 Advantages of ovarian tissue transplantation include that it can be performed in prepubertal girls and adolescents, can be performed at any point in the menstrual cycle, has the potential to save large numbers of oocytes, and may allow for spontaneous pregnancy in the future without in vitro fertilization or ovarian stimulation. Disadvantages include the need for surgery (typically by laparoscopy) to remove the tissue and risk of graft failure. There is also some evidence that oocyte quality may be compromised with lower than expected fertility rates even with IVF. Another very significant concern which may limit its usefulness for cancer patients include the possibility of contamination of ovarian tissue by malignant cells which has been reported with hematologic cancers and Ewing's sarcoma [21, 22].

 Patients undergoing ovarian tissue cryopreservation may still require future ovarian stimulation with gonadotropins and/or in vitro fertilization. Options that have been investigated to eliminate the risk of exposure to gonadotropins include in vitro maturation, (IVM) or in follicle maturation (IFM) of oocytes. These techniques require surgical removal of immature oocytes followed by in vitro exposure to gonadotropins to mature oocytes outside the body. There has been limited success with this approach utilizing immature oocytes aspirated during either the follicular or luteal phase of the menstrual cycle and matured in vitro. Although the survival rate is lower than with oocytes matured in vivo and vitrified, survival rates of 67.5% and clinical pregnancy rates of 20% have recently been reported [37]. Further data is needed to determine if this will be an effective treatment option for patients.

#### 3.2.5. Recent Developments in IVF for Cancer Patients

Until recently, preserving oocytes or embryos have required a delay in cancer treatment of up to 4–6 weeks to complete the IVF cycle. Traditional ovarian preparation for IVF required 10–14 days of ovarian stimulation with exogenous gonadotropins preceded by ovarian suppression with GnRH agonists for approximately 2 weeks to prevent premature ovulation. Medications were initiated in the luteal phase of the cycle which may add up to 3 additional weeks to the process depending on when the patient presents for treatment.

 Recent advances that include the development of GnRH antagonists have significantly decreased the interval from patient presentation to gamete cryopreservation. In contrast to GnRH agonists, GnRH antagonists immediately suppress pituitary release of FSH and LH and do not require the 10–14 days of administration prior to gonadotropin initiation. GnRH antagonists are initiated at approximately day 6 of gonadotropin stimulation which begins on day 2-3 of a menstrual cycle. This approach still requires awaiting menses prior to initiating gonadotropins but decreases the interval to oocyte retrieval compared to traditional IVF stimulation protocols.

A recent report of 3 patients initiating “random start IVF” evaluated the effectiveness of initiating GnRH antagonists at the time of patient presentation (menstrual cycle day 11, 14, and 17) rather than waiting for menses. This was then followed by the standard 10–14 days of ovarian stimulation and subsequent oocyte retrieval. The goal was to decrease the time to oocyte retrieval for breast cancer patients and resulted in a reasonable ovarian response with 7–10 embryos cryopreserved per patient [38]. This approach provides a significant advantage by decreasing total time for the IVF cycle, but further data is needed to determine its effectiveness compared to traditional IVF stimulation regimens.

In addition to the delay in cancer treatment, ovarian stimulation for IVF poses another theoretical risk to patients with hormonally responsive cancers. Ovarian stimulation with gonadotropins for IVF often results in supraphysiologic estradiol levels of over 2000 pg/mL compared to normal physiologic peak estradiol levels of 200–350 pg/mL. The high estradiol levels sustained during IVF treatment are a particular concern in women with estrogen receptor positive breast cancer. In the initial nonrandomized studies, stimulation protocols that include the selective estrogen receptor modulator tamoxifen or aromatase inhibitors such as letrozole administered during gonadotropin treatment have been shown to decrease estradiol level production while not decreasing overall oocyte numbers. Initial reassuring data indicates that this approach has not been shown to increase short-term cancer recurrences for breast cancer patients [39, 40]. 

Additionally, estradiol levels may be reduced after oocyte retrieval by the use of GnRH agonists to trigger ovulation instead of hCG. This has been shown to substantially reduce the risk of ovarian hyperstimulation in patients undergoing IVF by decreasing ovarian stimulation after retrieval. This has been evaluated in oocyte donors undergoing oocyte vitrification and has been shown in a retrospective study to result in similar numbers of oocytes retrieved. There was also no significant difference in the percentage of oocytes surviving thawing, oocyte fertilization, and pregnancy rates [41]. Further research is needed to determine if this will be beneficial in cancer patients undergoing oocyte or embryo banking but holds promise to further decrease any theoretical risks of breast cancer progression or recurrence as a result of ovarian stimulation.

#### 3.2.6. Additional Considerations

 Counseling of patients for future fertility should also include a discussion of alternative options including third-party reproduction. Third-party reproduction includes the use of either oocytes donated by another individual (either known or anonymously) as well as gestational carriers (“surrogates”) to carry a pregnancy. Oocyte donation may be utilized for patients without residual ovarian function after cancer treatment. Oocyte donation enables a patient to conceive and carry a pregnancy if she is unable to conceive with her own oocytes. Gestational carriers are most commonly used for patients that do not have a functional uterus to carry a pregnancy. They may also be considered for patients that are concerned about the recurrence of hormonally responsive tumors during pregnancy or any increased risks of pregnancy complications after cancer treatment. Adoption is also a consideration for family building but may be more difficult for cancer survivors than patients without a history of cancer [42]. 

 Unfortunately, a significant barrier for many cancer patients is the cost of fertility-preserving treatments. Insurance coverage is often not provided for these treatments that they are often considered “elective.” It has been argued that insurance companies should provide coverage for iatrogenic infertility as a result of cancer treatments similarly to coverage provided for other iatrogenic postcancer treatment conditions such as breast reconstruction after mastectomy and wigs for alopecia [43]. The average cost of fertility preservation for female cancer patients pursuing either embryo or oocyte cryopreservation is $8655 [44] and remains a barrier to access. Resources such as Fertile Hope's Sharing Hope Program can help patients and clinicians find centers with fertility preservation services as well as programs to provide financial assistance (http://www.fertilehope.org/). 

 Despite the concerns for patients including costs and potential risks, a followup survey of 28 cancer survivors who attempted fertility preservation found that 92.3% felt positively about their decision to undergo fertility preservation with only one patient, diagnosed with metastatic cancer shortly after oocyte retrieval, expressing regret [45]. The fact that patients with cancer recurrence may die and leave a minor child with one parent is an ethical concern. It has been suggested that it may be unethical to enable a woman to reproduce if she is expected to have a shortened lifespan. A review of fertility preservation and reproduction in cancer patients by the Ethics Committee of the Society for Reproductive Medicine stated that this concern may not be persuasive given that the risk of recurrence for many patients may not be excessively high, and the child may have a meaningful life despite the death of a parent [27].

### 3.3. Pregnancy after Cancer

#### 3.3.1. Conceiving after Cancer and the Risk of Pregnancy Complications

 The likelihood of conceiving after cancer treatments is dependent on the type of cancer, age at diagnosis, treatments with gonadotoxic agents including type and duration, and various other fertility factors. The chance for conception at best can only be estimated based on individual patient history and characteristics. It also appears that future fertility may also be influenced by gender. Overall, the likelihood of future children was found to be lower for female cancer survivors than male survivors either spontaneously or with fertility treatments [46]. When stratifying for age at diagnosis and estimating from probability charts, men with a cancer diagnosis prior to age 30 years had the highest overall chance of future parenthood (50%), followed by women diagnosed at age 30 years or younger (32%), then males diagnosed after age 30 years (12%), and then females diagnosed after age 30 years (<5%). For female patients the likelihood of pregnancy was dependent on the type of cancer and was highest for patients after uterine choriocarcinoma (65%), followed by lymphoma (23%) and malignant melanoma (22%), all other cancers (<5%).

 If pregnancy is established, there are several potential risks to a fetus conceived after cancer treatment. Both radiation and chemotherapy may induce chromosomal aberrations in oocytes that may theoretically increase the risk of birth defects and genetic disease in offspring. A review of studies evaluating the risk of malformations in offspring of breast cancer survivors did not report an increased risk of birth defects compared with controls [47]. It may be that any remaining pool of primordial follicles after treatments is unaffected by the prior treatment, and/or those oocytes that fertilize and develop into ongoing pregnancies are from a cohort of oocytes that do not demonstrate any carcinogenic effect. However, when considering the half-life of treatments and the duration of time for oocyte maturation, it has been recommended to delay pregnancy for at least 6 months [47] after treatment with chemotherapy and 12 months following completion of radiotherapy to minimize risks to offspring [48].

 Pregnancy complications and the subsequent risk to the fetus are another concern for cancer patients. A review of pregnancies in patients previously treated for breast cancer reported variable outcomes [47]. This meta-analysis evaluated 6 studies reporting birth outcome data after breast cancer compared to women without breast cancer. Four studies found no increased risk of any pregnancy complications; however, one study reported a higher risk of miscarriage and another reported no higher risk of miscarriage but a higher risk of cesarean section, preterm birth, low birth weight, delivery complications, and congenital abnormalities. The authors of the review suggest that although the large majority of births from women previously treated for breast cancer had no adverse effects, these women are at higher risk and may need careful monitoring until additional studies resolve the discrepancy in the data.

 A recent report of birth outcomes obtained from a childhood and adolescent cancer registry from 4 US regions has reported that infants born to female childhood cancer survivors were more likely to be preterm (RR 1.54), and to weigh less than 2500 g (RR 1.31). Although there appeared to be a higher risk during the pregnancy, there were no increased risks to the offspring of malformations, infant death, or altered sex ratio indicating no increased risk of germ cell mutagenicity [49]. A review of pregnancies postcancer diagnosis in adults indicated that subsequent pregnancy did not represent a major health risk for the mothers or children. In 678 pregnancies there were no increased risk of congenital malformations (OR 0.6) though pregnancies more often resulted in preterm delivery (OR 2.8), low birth weight (adds ratio 2.5), and cesarean section (OR 2.3) and were delivered on average 6 days earlier even after controlling for multiple births from patients utilizing fertility treatments to conceive [46].

 It is not clear whether the increased risks in pregnancy are related to the malignancy itself or the result of treatments such as radiation or chemotherapy. Several considerations exist in cancer patients that may affect the risk to a developing fetus including altered metabolism, nutrition deficiencies from malabsorption of nutrients, increased stress, and general overall decreased health. It is also possible that these patients might be subjectively viewed as higher risk by their physicians and are electively delivered earlier. In summary, it does appear that there may be an increased risk of preterm birth and associated neonatal complications for female cancer survivors, but the outcomes of the majority of pregnancies appear similar to noncancer patients.

#### 3.3.2. Risk of Transmission of Genetically Linked Cancers to Offspring

 Although there does not appear to be a definitive increased risk of congenital abnormalities for the offspring of female cancer patients, there is a concern over the transmission of genetically linked cancers. Hereditary cancers account for about 5% of all malignancies [50]. Most hereditary cancers follow an autosomal dominant mode of inheritance with the most common being hereditary nonpolyposis colorectal cancer, familial breast and ovarian cancer, neurofibromatosis type 1, familial retinoblastoma, multiple endocrine neoplasia type 2, and familial adenomatous polyposis. Fewer hereditary cancers have an autosomal recessive inheritance such as ataxia teleangiectatica and Fanconi anemia [51].

 Preimplantation genetic diagnosis (PGD) is a technique to screen embryos for genetically transmissible diseases prior to implantation. PGD involves removing one or more cells from an embryo after IVF and testing for predisposing mutations. PGD may be performed for genetic diseases where the gene has been identified and tested. PGD has been performed for all of the cancer predisposition syndromes mentioned previously in additional to several other less common susceptibility syndromes [52].

 Significant controversy exists over the ethical aspects of screening embryos for disease. A survey of 4,834 Americans in 2004 found that approximately 52% of women and 62% of men reported that they approved of PGD for screening embryos that had a tendency to develop a disease such as adult onset cancer [53]. A more recent survey of attendees at a national conference for individuals and families affected by hereditary breast and ovarian cancer reported that only 32% of participants had ever heard of PGD; however, 57% believed that it was an acceptable option for high-risk individuals and that patients should be given this information by their health-care provider [54]. The ASRM Ethics committee guidelines state that the concerns about the welfare of resulting offspring should not be cause for denying cancer patient's assistance and that preimplantation genetic diagnosis to avoid the birth of offspring with a high risk of inherited cancer is ethically acceptable. However, selection to avoid a genetic disease may not always be appropriate and factors such as the severity of the disease, the probability of its occurrence, and the age at onset should be considered.

#### 3.3.3. Pregnancy and Cancer Recurrence

 Cancer is diagnosed in one of every 118 pregnant women each year. There are several concerns for patients pursuing pregnancy after cancer treatment that may be dependent on the type of cancer and treatments. Concerns include the risk of cancer recurrence either during or after treatment, the possible increased risk of cancer recurrence secondary to pregnancy itself (breast cancer, endometrial cancer, and malignant melanoma), and the difficulty in detecting cancer during pregnancy (breast cancer and endometrial cancer).

 For most cancers, future pregnancy does not negatively impact the likelihood of recurrence. However, concern exists for several hormonally mediated cancers due to the consideration that the hormonal milieu of pregnancy may increase the risk of recurrence. The most common female tumors in reproductive age women that have been associated with hormonal mediators include breast cancer, endometrial cancer, and malignant melanoma. The most common cancer in women of childbearing age is breast cancer and is particularly concerning due to its clear association with hormonal markers. Patients with estrogen and/or progesterone receptor-positive tumors pose a particular challenge in counseling patients regarding recurrence risks during pregnancy and long-term overall recurrence risk.

 Approximately 2% of all breast cancers occur in women between 20 and 34 years of age and 11% in women between 35 and 45 years. Given the relatively young age at diagnosis and initial treatment, there is a risk of recurrence during the reproductive years. The overall risk of recurrence and timing of recurrence in the context of pregnancy is difficult to evaluate due to the complex associations with other predisposing factors such as age at diagnosis, prior pregnancy history, age at menarche, and family history. Additional considerations include whether or not pregnancy itself affects the long term survival for patients with breast cancer, and whether or not the timing of pregnancy affects any risk of recurrence.

 Initial studies in the 1980s and early 1990s indicated that there did not appear to be a difference in survival for time intervals from diagnosis to pregnancy [55–57]. In contrast, followup data indicated that patients that become pregnant in the first 3 months [58] or the first 6 months [59] of the initial breast cancer diagnosis may have an increased mortality. Clark et al. compared women that conceived within 6 months after a diagnosis of breast cancer to those patients that became pregnant between 6 and 24 months and more than 5 years after a diagnosis and found 5-year survival rates of 54%, 78%, and 100%, respectively. Another population-based study in 2006 showed a statistically nonsignificant increased mortality risk (RR 2.20, *P* = 0.58) for women diagnosed with breast cancer less than 6 months before pregnancy. However, if the interval was more than 2 years, the risk of death was reduced significantly (RR 0.48, *P* = 0.009) [60].

 A recent 2011 meta-analysis by Azim et al. has addressed the optimal timing of pregnancy for breast cancer patients. Five studies compared 353 patients who became pregnant within 6–24 months and after 2 years of a breast cancer diagnosis and found that pregnancy within 6–24 months or beyond 2 years did not have an effect on overall outcome. In summary, the data is controversial but it would appear prudent to advise waiting a minimum of 6 months after diagnosis to attempt pregnancy but more than 2 years is perhaps advisable and will depend on individual patient characteristics. For patients at higher risk of recurrence, a delay of 5 years or more may also be recommended.

 Interestingly, several studies have suggested that pregnancy is actually associated with a better long-term prognosis for breast cancer patients. The Azim et al. meta-analysis reviewed 14 studies of women who became pregnant after breast cancer and reported that 8 studies demonstrated a significant survival advantage while the remaining 6 showed a trend-favoring pregnancy but did not reach statistically significance [61], Once criticism of studies reporting an improved survival for patients with breast cancer is that they may have included selection bias referred to as the “healthy mother effect.”

 The “healthy mother effect” infers that women who become pregnant represent an overall healthier group of patients with perhaps a lower risk of disease relapse. The Azim et al. meta-analysis incorporated several sensitivity analyses to attempt to control for the “healthy mother effect” but still reported that women who got pregnant following breast cancer diagnosis had a 41% reduced risk of death compared to women who did not get pregnant and was most notable in patients with a history of node-negative disease. In a subgroup analysis, they compared the outcome of women with a history of breast cancer that became pregnant to breast cancer patients who did not get pregnant and did not find a difference in survival between the groups. A separate meta-analysis also controlling for the “healthy mother effect” also found similar results with a survival that was higher among early stage breast cancer patients compared to control (hazard ratio 0.51) for pregnancy that occurred at least 10 months after the diagnosis [62].

 The mechanism by which pregnancy may provide a protective effect is not clearly understood. It has been found that parous women have changes in expression of markers of disease recurrence including estrogen receptor alpha and beta (ER*α*, ER*β*) and human epidermal growth factor receptor 2 (HER2) for up to 10 years after pregnancy, which may provide protection from cancer recurrence [63]. Patterns of breast cancer recurrence have been evaluated with regard to estrogen receptor status in two randomized trials with 25 years of median followup. It was reported that most breast cancer recurrence occurred within the first 5–7 years in ER-negative after randomization while ER-positive patients had events spread through 10 years. Patients with ER-positive breast cancer generally receive 5 years of adjuvant hormonal therapy and are recommended to delay childbearing although some women may elect to interrupt hormonal therapy to conceive. Even for patients that may not be receiving adjuvant hormonal therapy, it is recommended that patients wait a minimum of 2 years following diagnosis to conceive due to a generally higher incidence of recurrence in the first 2 years after diagnosis [64]. Overall, available data support that pregnancy after breast cancer are safe for women at low risk of recurrence but the timing of pregnancy will depend on individual patient characteristics and estrogen receptor status.

 Although less common than breast cancer, malignant melanoma is a cancer with a peak incidence in the 30s and 40s resulting in a substantial number of women in their reproductive years interested in pursuing pregnancy after treatment. In contrast to breast cancer, there is limited evidence that hormonal mediators significantly influence this cancer. It has been noted that patients diagnosed during pregnancy often have a more rapid progression of their cancer, and estrogen-receptor proteins have been detected in tumor specimens. However, most studies have not found a statistical difference in 5-year survival rate for pregnant or nonpregnant patients. A study investigating endocrine ablation with procedures such as oophorectomy on patients with melanoma showed no benefit [65]. However, this author does suggest advising against future pregnancies for patients with nodal metastases or those who experienced tumor activation during a prior pregnancy. Others have recommended that all women with a history of melanoma avoid pregnancy for 3–5 years after treatment [66].

 Endometrial cancer is another hormonally mediated cancer as evidence by the fact that exposure to unopposed estrogen is a significant predisposing factor. Progesterone offers a protective effect on the endometrium but both estrogen and progesterone are elevated during gestation. Limited data is available regarding absolute risk of cancer progression or recurrence for patients with a history of endometrial cancer as most patients are treated with a hysterectomy. A small case series and literature review of 50 women reported data on women with early stage endometrial cancer treated with conservative hormonal treatment in lieu to a hysterectomy. There were 65 deliveries reported with 77 live births. No neonatal morbidity was noted but one of the 50 women died of her disease after delivery [67]. Another study found that 40% of patients treated with conservative treatment of progestin therapy conceived but had a 36% relapse rate of their cancer [68].

## 4. Conclusions

 Given the relatively high incidence of cancer in reproductive age women and improvements in 5-year survival, an increasing number of women are presenting for discussion of fertility preservation and pregnancy after cancer treatment. The ASCO published recommendations in 2006 on fertility preservation in cancer patients. These guidelines state that oncologists should address the possibility of infertility with cancer patients and be prepared to discuss possible fertility preservation options or refer the patient to a reproductive specialist.

 Part of the difficulty in counseling patients regarding the risk of infertility and/or subsequent pregnancy complications is that the risks are dependent on several factors. These risks include the dose and duration of treatment, other risk factors for infertility, the age of the patient, and the patient's baseline ovarian reserve at the time of initiation of treatment. Advancements in ovarian reserve testing may help counsel patients about the impact of their cancer treatments on fertility and chances for future pregnancy.

 Fertility preservation is a rapidly evolving field that includes medical and surgical treatments to decrease the impact of cancer treatments on future fertility. Ongoing trials will address the effectiveness of GnRH agonists in protecting ovarian reserve. Several technologies exist to help preserve future fertility including embryo cryopreservation, oocyte, and ovarian tissue cryopreservation. Embryo cryopreservation is currently the only recommended method of gamete preservation, but recent advances in oocyte vitrification may increase the utility of this treatment for cancer patients. Additionally, PGD may decrease the risk of disease transmission of hereditary cancer syndromes. The risk to the patient of IVF may also be decreased with recent advances in IVF stimulation protocols.

 There may be an increased risk of preterm birth and associated neonatal complications for female cancer survivors, but the outcomes of the majority of pregnancies appear similar to noncancer patients. It is not clear whether the increased risks in pregnancy are related to the malignancy itself or the result of treatments such as radiation or chemotherapy. Also, the risk of disease recurrence will depend on several factors, but for most cancers the risk of recurrence is not increased secondary to pregnancy. Overall, pregnancy appears safe for most patients after cancer treatment but will depend on individual patient characteristics.
